# Moving Object Detection Based on Optical Flow Estimation and a Gaussian Mixture Model for Advanced Driver Assistance Systems

**DOI:** 10.3390/s19143217

**Published:** 2019-07-22

**Authors:** Jaechan Cho, Yongchul Jung, Dong-Sun Kim, Seongjoo Lee, Yunho Jung

**Affiliations:** 1 School of Electronics and Information Engineering, Korea Aerospace University, Goyang-si 10540, Korea; 2 Korea Electronics Technology Institute, Seongnam-si 463-816, Korea; 3 Department of Information and Communication Engineering, Sejong University, Seoul 143-747, Korea

**Keywords:** ADAS, background subtraction, FPGA, moving object detection, optical flow estimation

## Abstract

Most approaches for moving object detection (MOD) based on computer vision are limited to stationary camera environments. In advanced driver assistance systems (ADAS), however, ego-motion is added to image frames owing to the use of a moving camera. This results in mixed motion in the image frames and makes it difficult to classify target objects and background. In this paper, we propose an efficient MOD algorithm that can cope with moving camera environments. In addition, we present a hardware design and implementation results for the real-time processing of the proposed algorithm. The proposed moving object detector was designed using hardware description language (HDL) and its real-time performance was evaluated using an FPGA based test system. Experimental results demonstrate that our design achieves better detection performance than existing MOD systems. The proposed moving object detector was implemented with 13.2K logic slices, 104 DSP48s, and 163 BRAM and can support real-time processing of 30 fps at an operating frequency of 200 MHz.

## 1. Introduction

Advanced driver assistance systems (ADAS) represent the most popular field in the automotive industry and have become a key technology for modern vehicle safety and driving comfort [1,2]. The most commonly used ADAS techniques include adaptive cruise control, collision warning and lane change assistance. Collision warning systems are one of the major applications of ADAS and their task is to inform drivers of obstacles around the vehicle by giving visual, aural, or tactile feedback [3,4]. Reliable moving object detection (MOD) technology is an essential part of collision warning systems and various sensor-based techniques have been proposed, such as vision-, lidar- and radar-based techniques [5,6]. Since vision-based MOD technology is relatively more intuitive and cheaper than active sensor techniques, such as radar and lidar, many vision-based algorithms have been proposed [7,8,9,10,11,12,13,14,15,16,17,18,19,20,21,22,23,24,25,26,27,28,29].

However, several vision-based MOD algorithms assume input image frames to be captured by stationary camera, because detection accuracy degrades for moving cameras [8,9,10,11,12,13,14]. Although methods for generating background models using smart cameras have also been proposed, they suffer from a limitation in that they can only be used in stationary camera environments [15,16,17]. Without proper distinction, the mixed motion between background and foreground objects that is caused by moving cameras is hard to distinguish. Using a moving camera is inevitable in vehicle environments and thus an efficient algorithm that can cope with this fact is needed.

For this reason, several algorithms that allow for MOD in the image frames obtained by moving cameras have recently been proposed [18,19,20,21,22,23,24,25,26,27,28,29]. Some of these methods employ advanced statistical models or outlier detection techniques to estimate the background [18,19,20,21,22,23,24]. In Reference [22], background subtraction based on a 2.5D background model was proposed, which could be used to successfully detect moving objects in complex scenes. In Reference [23], a fast and effective MOD algorithm based on global motion compensation and adaptive background modelling was presented, which supports real-time object detection on a moving camera. The method proposed in Reference [24] introduces a novel background modelling approach based on dynamic reverse analysis (DRA). This approach can handle illumination variations, occlusions and camera instability. A comprehensive overview of the recent efforts made for dealing with illumination variations and occlusions can be found in Reference [25]. Recently, algorithms that rely on deep neural networks (DNNs) have also been proposed [26,27,28]. In the method proposed in Reference [28], a single CNN is trained using stacked depth-wise image-background pairs, and its output is enhanced via post processing. However, these algorithms may not be suitable for ADAS applications with limited power consumption and available area because of the associated high computational complexity and massive memory requirements. In addition, algorithms based on learning methods cannot deal with unexpected situations because the image frames used for learning are restricted to specific cases [29]. In particular, the use of moving cameras results in a wide variety of possible situations, which seriously degrades MOD performance.

On the other hand, a technique for compensating the camera motion, referred to as ego-motion, without relying on learning methods has been proposed [29]. The MOD algorithm proposed in Reference [29] estimates the flow vector of each pixel via a Lucas Kanade (LK)-based optical flow estimation (OFE) algorithm and analyzes the histogram of the flow vectors to estimate the ego-motion. Then, the background model is compensated using the estimated ego-motion. The compensated background model is used in the MOD process to separate out the mixed motion between the background and foreground objects. This algorithm can prevent many false positives and shows good precision in moving camera environments. However, there is a problem in that its performance in terms of recall is degraded. Therefore, its applications are limited to backup collision intervention (BCI), which consists in detecting obstacles behind the vehicle, because such tasks require relatively slow camera motion, as explained in Reference [29]. This drawback arises owing to the use of LK-based OFE for ego-motion estimation. LK-based OFE is a local method for estimating flow vectors based on the assumption that the motion of a local region is the same within itself. A limitation of this method is that it cannot find the correct flow vectors in regions where the brightness pattern is uniform. Therefore, inaccurate flow vectors estimated in the background regions with uniform brightness patterns have an adverse effect on ego-motion estimation.

In order to overcome the problems associated with the use of local methods, such as LK-based OFE, Horn and Schunk (HS) proposed a global method for defining the energy function of an entire image frame and for estimating the flow vectors while minimizing this energy function [30]. However, this method cannot cope with sudden changes in brightness and various algorithms have been proposed to solve this problem [31,32,33]. Among them, the Brox algorithm shows robustness to changes in brightness by extending the conventional OFE brightness constancy assumption to a gradient constancy assumption. It also shows higher accuracy than other algorithms in vehicle environments.

In this paper, we propose an efficient algorithm for finding moving objects after more precisely estimating and compensating ego-motion using the Brox OFE algorithm. In addition, we present a hardware structure design and its results for real-time processing tasks. The remainder of this paper is organized as follows: Section 2 explains the Brox OFE algorithm and the Gaussian mixture model (GMM). Section 3 presents the proposed MOD algorithm, and Section 4 describes the hardware architecture of the proposed moving object detector. Section 5 presents the results for an FPGA implementation of the proposed moving object detector and performance evaluation results. Finally, Section 6 concludes the paper.

## 2. Related Work

### 2.1. Brox Optical Flow Estimation

Let $x={\left(x,y,t\right)}^{T}$ and $w={\left(u,v,1\right)}^{T}$, where x denote pixel information at time *t* and w represents the estimated flow vector. Then, global deviations from the grey value constancy assumption and the gradient constancy assumption can be measured by the energy as

(1) $$E_{Data}\left(u,v\right)\;=\;\int_{\Omega }\psi \left({\left|I(x+w)\;-\;I(x)\right|}^{2}\;+\;\gamma \;{\left|\nabla I(x+w)\;-\;\nabla I(x)\right|}^{2}\right)\;\mathrm{dx},$$

where γ is a weight between both assumptions. An increasing concave function $\psi \left(s^{2}\right)=\sqrt{\left(s^{2}+\epsilon^{2}\right)}$ is applied, leading to a robust energy estimation [31], where ϵ is small positive constant that is typically set as 0.001. Smoothness describes the model’s assumption of a piecewise smooth flow field and can be expressed as follows:

(2) $$E_{Smooth}\left(u,v\right)\;=\;\int_{\Omega }\psi \left({\left|\nabla_{3}u\right|}^{2}+{\left|\nabla_{3}v\right|}^{2}\right)\;\mathrm{dx}.$$

The spatio-temporal gradient $\nabla_{3}={\left(\delta_{x},\delta_{y},\delta_{t}\right)}^{T}$ indicates that a spatio-temporal smoothness assumption is involved. The total energy is the weighted sum of (1) and (2), and it can be depicted by

(3) $$E(u,v)\;=\;E_{Data}\;+\alpha E_{Smooth},$$

where α is a regularization parameter. The value of (u,v) that minimizes the total energy is the optimal flow vector.

The Euler–Lagrange equation is applied to (3) to find the optimal solution. To linearize the equation, Brox applied a numerical approximation. A multi-scale approach, also called a pyramid, and an inner iteration loop are applied to suppress the non-linearity of the remaining ψ term [31]. Then, the final optimal equation is converted into matrix form for all the pixels within an image frame as follows:

(4) $$\begin{array}{c} {\left(\psi^{\prime }\right)}_{Data}^{k,l}\cdot \;\left(\begin{array}{c} I_{x}^{k}I_{x}^{k}\;+\;\gamma \left(I_{xx}^{k}I_{xx}^{k}\;+\;I_{xy}^{k}I_{xy}^{k}\right)\\ I_{y}^{k}I_{x}^{k}\;+\;\gamma \left(I_{yy}^{k}I_{yx}^{k}\;+\;I_{xy}^{k}I_{xy}^{k}\right) \end{array}\right.\left.\begin{array}{c} \;I_{x}^{k}I_{y}^{k}\;+\;\gamma \left(I_{xx}^{k}I_{xy}^{k}\;+\;I_{xy}^{k}I_{yy}^{k}\right)\\ I_{y}^{k}I_{y}^{k}\;+\;\gamma \left(I_{yy}^{k}I_{yy}^{k}\;+\;I_{xy}^{k}I_{xy}^{k}\right) \end{array}\right)\;\cdot \;\left(\begin{array}{c} du^{k,l+1}\\ dv^{k,l+1} \end{array}\right)\\ \;\;\;\;\;=-\;{\left(\psi^{\prime }\right)}_{Data}^{k,l}\cdot \;\left(\begin{array}{c} I_{x}^{k}I_{t}^{k}\;+\;\gamma \left(I_{xx}^{k}I_{xt}^{k}\;+\;I_{xy}^{k}I_{yt}^{k}\right)\\ I_{y}^{k}I_{t}^{k}\;+\;\gamma \left(I_{yy}^{k}I_{yt}^{k}\;+\;I_{xy}^{k}I_{xt}^{k}\right) \end{array}\right)+\;\alpha \cdot \left(\begin{array}{c} \mathrm{div}({\left(\psi^{\prime }\right)}_{Smooth}^{k,l}\;\nabla_{3}\;\left(u^{k}\;+\;du^{k,l}\right))\\ \mathrm{div}({\left(\psi^{\prime }\right)}_{Smooth}^{k,l}\;\nabla_{3}\;\left(v^{k}\;+\;dv^{k,l}\right)) \end{array}\right), \end{array}$$

where $I_{x},I_{y},I_{t},I_{xx},I_{xy},I_{xt},$ and $I_{yt}$ represent the gradient for each direction; *k* is the pyramid loop; and *l* indicates the iteration loop for linearization in each pyramid loop [31]. In (4), ${\left(\psi^{\prime }\right)}_{Data}$ and ${\left(\psi^{\prime }\right)}_{Smooth}$ are obtained as follows:

(5) $$\begin{array}{ll} {\left(\psi^{\prime }\right)}_{Data}^{k,l} & =\;\;\psi^{\prime }\left({\left(I_{t}^{k}\;+\;I_{x}^{k}du^{k,l}\;+\;I_{y}^{k}dv^{k,l}\right)}^{2}\right.\;\\ & +\gamma \left({\left(I_{xt}^{k}\;+\;I_{xx}^{k}du^{k,l}\;+\;I_{xy}^{k}dv^{k,l}\right)}^{2}\;\left.\left.+\;{\left(I_{yt}^{k}\;+\;I_{xy}^{k}du^{k,l}\;+\;I_{yy}^{k}dv^{k,l}\right)}^{2}\right)\right)\;,\right. \end{array}\;$$

(6) $${\left(\psi^{\prime }\right)}_{Smooth}^{k,l}=\;\;\psi^{\prime }\left({\left|\nabla_{3}\left(u^{k}\;+\;du^{k,l}\right)\right|}^{2}\;+\;{\left|\nabla_{3}\left(v^{k}\;+\;dv^{k,l}\right)\right|}^{2}\right).$$

If $du^{k,l+1}$ and $dv^{k,l+1}$ in the left-hand side of (4) are denoted as vector **w**, the remaining term as matrix **A**, and the reight-hand side of (4) as vector **b**, then, (4) can be expressed as Aw=b, and the final solution for d can be calculated as

(7) $$w=A^{-1}b.$$

### 2.2. Gaussian Mixture Model

The GMM algorithm, which was proposed by Stauffer and Grimson [10], estimates the background image by employing a statistical model of intensity for each pixel in the image frame. Several works have been carried out to improve its performance, and the GMM has been widely adopted as a basic framework for generating background models [11,12,13,14]. Since the GMM algorithm available in the open-source computer vision software library (OpenCV) [34] has been optimized in terms of performance and complexity, it has been adopted in various FPGA implementations [35,36,37] and is also applied in our proposed algorithm.

The GMM algorithm is composed by a mixture of *n* Gaussian distributions represented by three parameters: weight (*w*), mean (μ) and variance ($\sigma^{2}$). The Gaussian distributions of each pixel have different parameters and change for each image frame. Therefore, these parameters are defined by three indices, namely i,n, and t, where *i* is the index for pixel intensity, *n* denotes the index for the Gaussian distributions, and *t* is an index that refers to the time of the considered frame.

These parameters are updated differently depending on the match condition, which indicates whether a pixel is suitable for the background model. The match condition is checked against the *n* Gaussian distributions that model the pixel and is given by

(8) $$m_{n}=1,\;\;\;\;\;\;\;\;if\;{|i_{t}-\mu_{n,t}|}^{2}\;\leq \;D^{2}\cdot \sigma_{n,t}^{2},$$

where *D* is a threshold whose value was experimentally chosen to be equal to 2.5. The background model of each pixel is generated in a grey scale ranging from 0 to 255 using the mean and the weight of the Gaussian model as follows:

(9) $$B_{t}=\sum_{k=1}^{K}w_{n,t}\cdot \mu_{n,t}.$$

## 3. Proposed Moving Object Detection Algorithm

**Figure 1:**
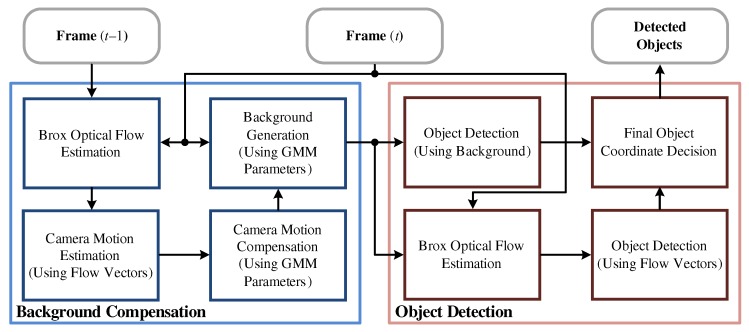
Overall scheme of the proposed moving object detection (MOD) algorithm.

Since the image frames obtained from moving cameras contain the motion of both the background and the objects, the proposed MOD algorithm performs a background compensation process before detecting objects, as shown in Figure 1. The background compensation process estimates the ego-motion and generates the compensated background. Then, the object detection process extracts the object coordinates using the current image frame and the compensated background. To minimize noise, two detection methods are performed and the final object coordinates are determined by cross-checking the results. After matching the resolution scales for two output coordinates, the final result is confirmed through an intersection operation.

### 3.1. Background Compensation

In order to estimate the ego-motion, the Brox OFE algorithm is applied to two consecutive frames to extract the flow vectors. Since the matrix inversion of **A** in (7) requires computation time proportionally to the image frame size and **A** is a Hermitian positive-definite matrix, we apply Cholesky factorization to **A**:

(10) $$A={\mathrm{LL}}^{T},$$

where **L** is a lower triangular matrix. It is much easier to compute the inverse of a triangular matrix [38] and the inverse of the original matrix can be computed by simply multiplying the two inverses as follows:

(11) $$A^{-1}={\left(L^{-1}\right)}^{T}\left(L^{-1}\right).$$

Then, the final flow vector **w** is calculated as

(12) $$w={\left(L^{-1}\right)}^{T}\left(L^{-1}\right)b.$$

The extracted flow vector for each pixel represents the motion of the pixel between consecutive frames. The pixels in the background region have a relatively slow motion compared with the object region. In addition, background pixels occupy a larger area in the image frame than objects and exhibit similar motion. Therefore, the most frequent flow vector with slow motion is regarded as the ego-motion, which can be extracted via histogram analysis. Then, the previously derived background model is compensated using the extracted ego-motion. In this approach, the background model is derived via the GMM algorithm and stored as GMM parameters.

After estimating the ego-motion, the entire GMM parameters can be shifted back along the determined motion, resulting in a compensated background model. First, the ego-motions $e_{dx}$ and $e_{dy}$ in the x-axis and y-axis directions, respectively, are divided into an integer part and a fractional part. The integer parts $e_{dx}^{i}$ and $e_{dy}^{i}$ can be obtained by rounding up $e_{dx}$ and $e_{dy}$ respectively, whereas the fractional parts $e_{dx}^{f}$ and $e_{dy}^{f}$ are computed as

(13) $$e_{dx}^{f}=e_{dx}-e_{dx}^{i},$$

(14) $$e_{dy}^{f}=e_{dy}-e_{dy}^{i}.$$

**Figure 2:**
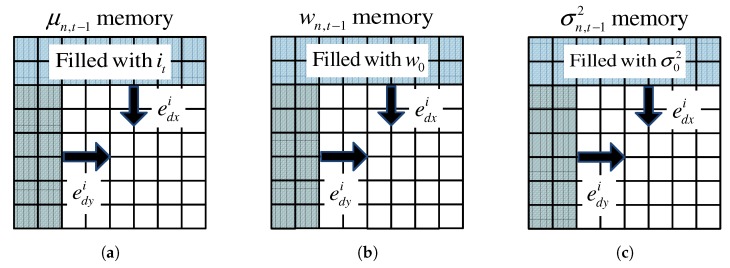
Compensation for the integer parts of the ego-motion. The shaded region denotes empty space generated by the shift operation: (**a**) $\mu_{n,t-1}$ memory; (**b**) $w_{n,t-1}$ memory; (**c**) $\sigma_{n,t-1}^{2}$ memory.

Then, the GMM parameters $w_{n,t-1},\mu_{n,t-1}$ and $\sigma_{n,t-1}^{2}$ are shifted by $e_{dx}^{i}$ and $e_{dy}^{i}$ as shown in Figure 2. The empty space caused by the shift operation for $\mu_{n,t-1}$ is filled with the $i_{t}$ of the same position, as shown in Figure 2a. On the other hand, the empty spaces are also filled with the pre-defined values $w_{o}$ and $\sigma_{o}^{2}$ for $w_{k,t}$ and $\sigma_{k,t}^{2}$, respectively, as depicted in Figure 2b,c.

Afterwards, the GMM parameters are interpolated using the fractional parts of the ego-motion, namely $e_{dx}^{f}$ and $e_{dy}^{f}$. The interpolation for $e_{dx}^{f}$ is performed in the x-axis direction as shown in (15)–(17) and the same is done for $e_{dy}^{f}$ in the y-axis direction, as presented in (18)–(20):

(15) $$w_{n,t-1}^{x}\left(x,y\right)=e_{dx}^{f}\cdot w_{n,t-1}\left(x,y\right)+\left(1-e_{dx}^{f}\right)\cdot w_{n,t-1}\left(x+1,y\right),$$

(16) $$\mu_{n,t-1}^{x}\left(x,y\right)=e_{dx}^{f}\cdot \mu_{n,t-1}\left(x,y\right)+\left(1-e_{dx}^{f}\right)\cdot \mu_{n,t-1}\left(x+1,y\right),$$

(17) $$\sigma_{n,t-1}^{x}\left(x,y\right)=e_{dx}^{f}\cdot \sigma_{n,t-1}^{2}\left(x,y\right)+\left(1-e_{dx}^{f}\right)\cdot \sigma_{n,t-1}^{2}\left(x+1,y\right),$$

(18) $$w_{n,t}\left(x,y\right)=e_{dy}^{f}\cdot w_{n,t-1}^{x}\left(x,y\right)+\left(1-e_{dy}^{f}\right)\cdot w_{n,t-1}^{x}\left(x,y+1\right),$$

(19) $$\mu_{n,t}\left(x,y\right)=e_{dy}^{f}\cdot \mu_{n,t-1}^{x}\left(x,y\right)+\left(1-e_{dy}^{f}\right)\cdot \mu_{n,t-1}^{x}\left(x,y+1\right),$$

(20) $$\sigma_{n,t}^{2}\left(x,y\right)=e_{dy}^{f}\cdot \sigma_{n,t-1}^{x}\left(x,y\right)+\left(1-e_{dy}^{f}\right)\cdot \sigma_{n,t-1}^{x}\left(x,y+1\right).$$

After the compensation process is complete, the new GMM parameters are updated through the GMM algorithm. Then, the final background model, that is, the compensated background model is generated via (9) using $w_{n,t},\mu_{n,t},$ and $\sigma_{n,t}^{2}$.

### 3.2. Object Detection

To extract object coordinates in an image frame using the compensated background model, the proposed MOD algorithm performs a background subtraction operation first, followed by Brox OFE. Background subtraction consists in separating moving objects from stationary background images. If the difference between the compensated background model and the current frame is larger than the threshold, it is classified as a moving object and the rest is classified as background. Although this approach can effectively detect objects, such a simple comparison results in false positives.

In order to solve this problem, Brox OFE between the compensated background and the current frame is performed to extract the flow vectors of all pixels. The extracted flow vectors that have different magnitude and direction from those of the background can be grouped into objects. To group the object regions from the overall vectors in a frame, a proper threshold should be determined. This threshold has to be chosen for each frame by considering the distribution of the flow vectors. Since we derived the detection results via background subtraction, the final detection results are determined via cross-checking with both sets of results to reduce the number of false positives. A median filter on the results is applied to remove relatively small objects, such as those caused by noise.

## 4. Hardware Architecture Design

**Figure 3:**
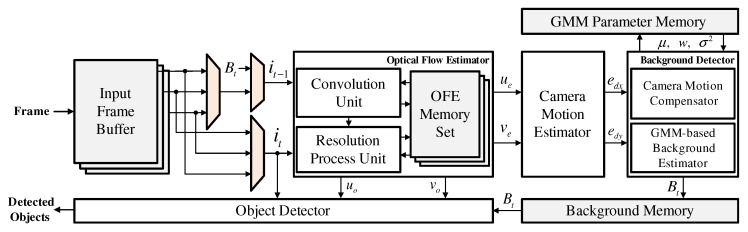
Block diagram of the proposed moving object detector.

In this section, we present the hardware architecture of the proposed moving object detector for real-time processing. Figure 3 shows a block diagram of the proposed moving object detector, which consists of an optical flow estimator, a camera motion estimator, a background detector and an object detector. The data stream of the image frame, which enters from the external camera module, is stored in the input frame buffers. Then, the pixel intensities of two consecutive frames $i_{t-1}$ and $i_{t}$ are selected from these buffers to estimate flow vectors $u_{e}$ and $v_{e}$ for, in turn, estimating the ego-motion via the optical flow estimator. The histogram statistics of these flow vectors are analyzed by the camera motion estimator to extract the ego-motions $e_{dx}$ and $e_{dy}$. In order to generate the compensated background $B_{t}$, the background detector shifts the GMM parameters according to the estimated ego-motions, as explained in Section 3.1, and updates the corresponding parameters by applying the GMM algorithm. Using $B_{t}$ and the pixel intensities of current frame it, the object detector performs background subtraction, and the optical flow estimator simultaneously extracts new flow vectors $u_{o}$ and $v_{o}$. These flow vectors are used by the object detector to classify the object region. Finally, the object coordinates are generated by combining the two sets of detection results.

### 4.1. Optical Flow Estimator

**Figure 4:**
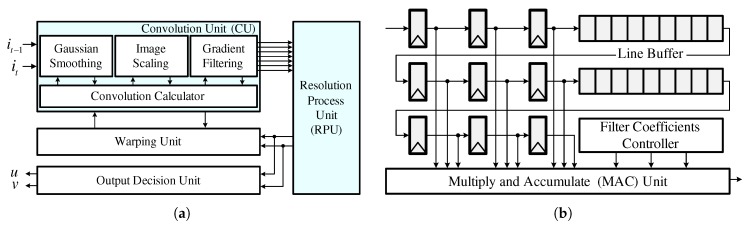
Hardware structure: (**a**) optical flow estimator; (**b**) convolution calculator.

The optical flow estimator shown in Figure 4a is composed of a convolution unit (CU) for pre-processing, a resolution process unit (RPU) for computing the solution of the Euler-Lagrange equation, a warping unit, and an output decision unit. Since a multi-scale approach (also called pyramid) is required, the input frames are scaled to a lower resolution after Gaussian smoothing. Then, a gradient filtering module calculates $I_{x},I_{y},I_{t},I_{xx},I_{xy},I_{xt},$ and $I_{yt}$ using the scaled image frames. The Gaussian smoothing, image scaling, and gradient filtering operations are grouped into the CU and have a shared structure in the convolution calculator to reduce hardware complexity. This is possible because they perform similar image filtering operations. Employing this shared structure reduces the number of multipliers by ten, that of adders by five, and that of line buffers by four, as shown in Figure 4b. After gradient filtering is complete, the RPU computes (4) to extract the flow vectors. Then, the warping unit generates higher resolution image frames using the previously scaled data and extracted flow vectors. The overall operation of the optical flow estimator is repeated during the pyramid loop.

**Figure 5:**
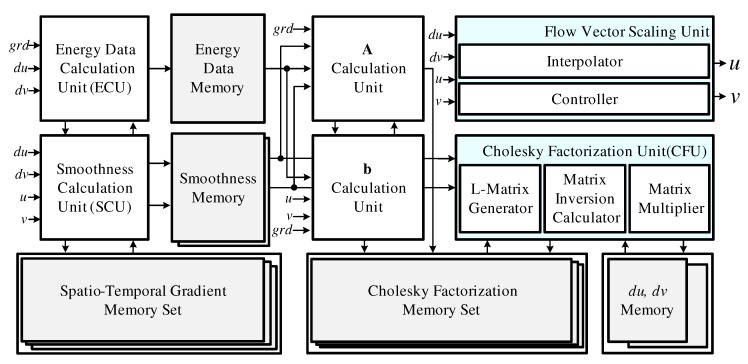
Block diagram of the resolution process unit.

Figure 5 shows the structure of the RPU, which consists of an energy data calculation unit (ECU), a smoothness calculation unit (SCU), calculation units for **A** and **b**, a Cholesky factorization unit (CFU), and a flow vector scaling unit. The ECU and SCU simultaneously compute the $\psi_{Data}$ and $\psi_{Smooth}$ terms via (5) and (6). Then, the calculation units for **A** and **b** use the results of each operation to calculate **A** and **b** in the final matrix equation as shown in (4). Since similar calculations are repeated in (4), (5), and (6), we employed a shared structure to reduce the number of operators and memory requirements. The CFU factorizes **A** as lower triangular matrixes **L** and $L^{T}$ and performs matrix inversion. These operations require excessive memory access, which depends on image frame size. Excessive memory access results in high power consumption and makes real-time processing impossible. Therefore, we apply a shift register bank, which can reduce the number of memory access operations by 95.75%.

### 4.2. Camera Motion Estimator

**Figure 6:**
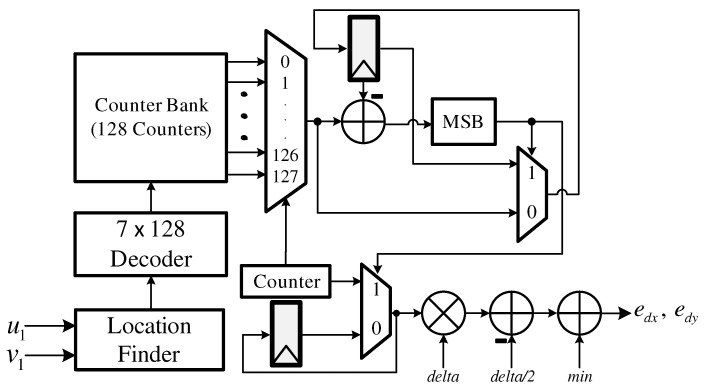
Hardware structure of the camera motion estimator.

Figure 6 depicts the camera motion estimator, which is composed of a location finder, a 7 × 128 decoder, a counter bank, and some calculators. The designed camera motion estimator analyzes the histogram of the flow vectors. The histogram is generated by dividing the entire range of the flow vectors into a series of intervals and then counting how many vectors fall into each interval. We divide the entire range into 128 intervals, considering the trade-off between hardware complexity and performance. Histogram analysis is performed using the counter circuits and the control signal of each counter is generated by the location finder and the 7 × 128 decoder. Finally, $e_{dx}$ and $e_{dy}$ are extracted by finding the maximum count value.

### 4.3. Background Detector

**Figure 7:**
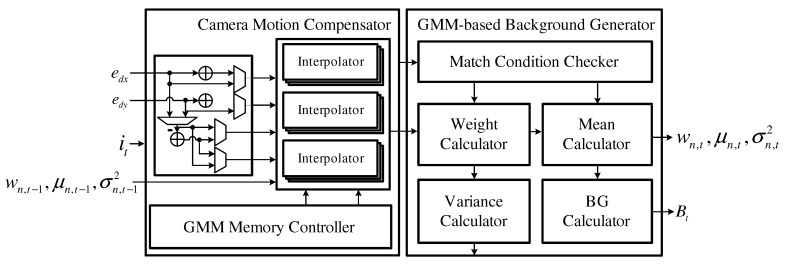
Block diagram of the background detector.

The background detector, which is shown in Figure 7, is composed of a camera motion compensator that performs compensation for the GMM parameters using $e_{dx}$ and $e_{dy}$ and a GMM-based background estimator that updates the GMM parameters and estimates the background $B_{t}$. The camera motion compensator performs a shift operation with integer parts $e_{dx}^{i}$ and $e_{dy}^{i}$ and then interpolates the GMM parameters using fractional parts $e_{dx}^{f}$ and $e_{dy}^{f}$. The GMM-based background estimator updates the compensated GMM parameters according to the current image frame and simultaneously estimates the background $B_{t}$.

### 4.4. Object Detector

**Figure 8:**
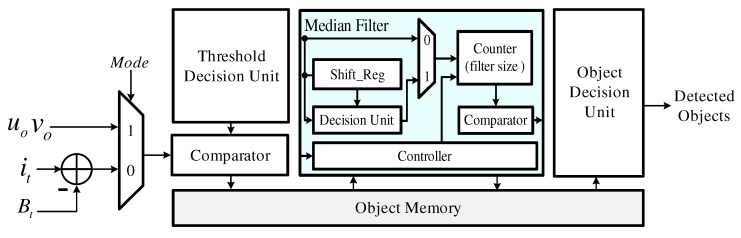
Block diagram of the object detector.

Figure 8 shows the object detector, which consists of a background subtractor, a threshold decision unit, an object memory, a median filter and an object decision unit. First, the absolute value of the difference between $i_{t}$ and $B_{t}$ is provided to the comparator and object candidates are generated by comparing this value with an experimentally determined threshold value. These background subtraction results are stored in object memory. Afterwards, the comparator generates object candidates using the flow vectors and the threshold which is determined according to the distribution of these vectors. The generated object candidates are also stored in the object memory, and median filtering is performed. Finally, the coordinates of the objects are detected by cross-checking the two sets of results in the object decision unit.

## 5. Experimental Results

### 5.1. FPGA Implementation

The proposed moving object detector was designed using hardware description language (HDL) and implemented on a Xilinx Virtex5 FPGA device. As a result, the proposed moving object detector was implemented with 13.2K logic slices, 104 DSP48s, and 163 BRAM, as shown in Table 1. The comparison results between the proposed GMM-based background generator and previous GMM implementations [35,36] are presented in Table 2. The GMM-based background generator employed in the proposed design has a similar complexity to the method presented in Reference [36] and can be implemented using less resources than that presented in Reference [35].

Since the final object coordinates are generated at intervals of 6.67M clock cycles for an image resolution of 640 × 480, we confirmed that real-time processing at 30 fps is possible using an FPGA test system at 200 MHz. The total number of clock cycles is proportional to the resolution of the input image. Table 3 shows comparison results in terms of processing speed between this work and other MOD scheme that can perform real-time operation on moving camera environments. The results confirm that the proposed system is significantly faster in terms of processing speed (fps) than other schemes that can support real-time processing.

**Figure 9:**
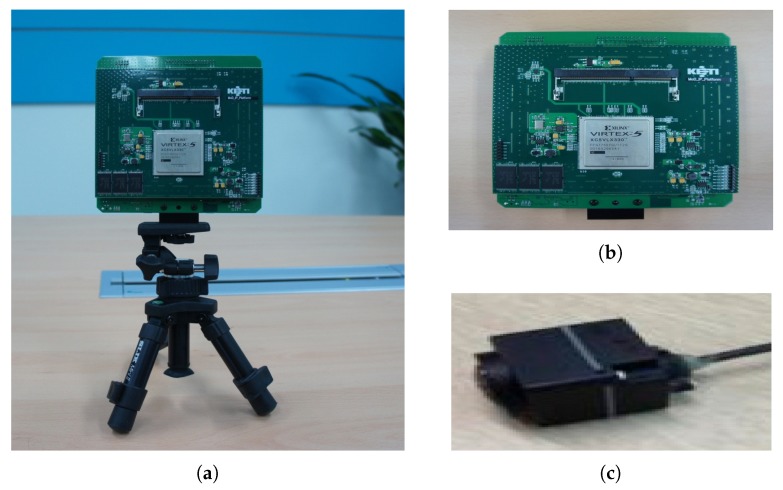
FPGA test platform: (**a**) test environment; (**b**) Xilinx Virtex-5 FPGA based evaluation board; (**c**) 640 × 480 resolution camera.

In order to evaluate the performance of the proposed moving object detector in actual vehicle environment, an FPGA test platform was constructed and is shown in Figure 9. This verification platform included an FPGA device with the proposed moving object detector, a 640 × 480-resolution camera and an HDMI recorder.

### 5.2. Performance Evaluation

MOD performance metrics, namely precision ($P_{r}$), recall ($R_{e}$), and F-measure ($F_{m}$), were used to carry out a numerical comparison between existing and proposed algorithms and the proposed algorithm. These metrics are defined as follows:

(21) $$P_{r}=\frac{TP}{\left(TP+FP\right)},$$

(22) $$R_{e}=\frac{TP}{\left(TP+FN\right)},$$

(23) $$F_{m}=\frac{2\cdot P_{r}\cdot R_{e}}{\left(P_{r}+R_{e}\right)}.$$

True positives (TP) represents the total number of actual object pixels that are recognized as an object and false negatives (FN) denotes the total number of actual object pixels that are erroneously recognized as background. False positives (FP) represents the total number of background pixels that are recognized as an object. Therefore, $P_{r}$ quantifies the precision of actual object pixels among all the pixels recognized by the algorithm as objects and $R_{e}$ quantifies the detection rate as the ratio of pixels recognized by the algorithm as object to actual object pixels.

Table 4 shows the results obtained by applying existing MOD algorithms and the proposed moving object detector to 200 consecutive image samples with three vehicles moving to the right [39]. Two rank-constrained models [19,20] exhibited excellent recall performance, but their precision was low, which would give the driver many false alarms. Although the algorithm presented in Reference [29] exhibited a higher precision than those of References [19,20], its recall performance was lower. In contrast, the proposed moving object detector exhibited the same precision as the algorithm from Reference [29], minimized the number of false alarms, and had a recall performance of 95%, which is 17% higher than the algorithm from Reference [29].

**Figure 10:**
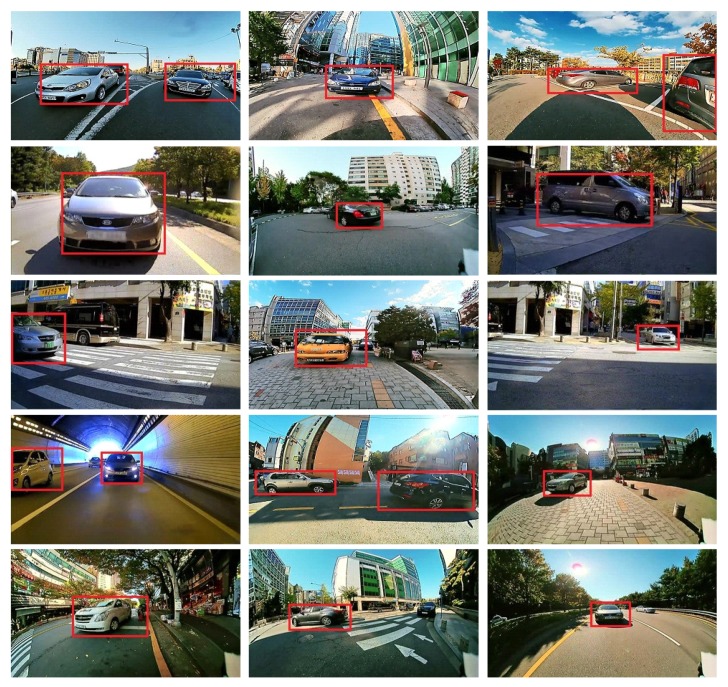
MOD performance of the proposed moving object detector.

Figure 10 shows examples of the experimental results obtained after applying the proposed moving object detector to the image samples taken from a vehicle equipped with the FPGA platform shown in Figure 9. As can be seen from Figure 10, the proposed algorithm exhibited good object detection performance in a vehicle environment, and we confirmed that false positives hardly ever happened.

## 6. Conclusions

In this paper, we proposed a novel MOD algorithm, which can operate in moving camera environments. In addition, an area-efficient hardware design for the proposed algorithm was presented for real-time processing. Experimental results demonstrate the overall improvements achieved using the proposed algorithm in terms of precision, recall and F-measure, which are important features for ADAS applications. The proposed moving object detector was implemented with 13.2 K logic slices, 104 DSP48s, and 163 BRAM and an FPGA test platform was constructed for verification in a vehicle environment. Through this verification, we confirmed that the proposed moving object detector achieved higher accuracy than existing MOD algorithms and that it can support real-time processing at 30 fps and an operating frequency of 200 MHz.

## Figures and Tables

**Table 1:** Implementation results for the proposed moving object detector.

Block	FPGA Logic Slices (/51840)	DSP48s (/192)	Block RAM (/972)
Optical flow estimator	12,312	96	108
Camera motion estimator	326	1	0
Background detector	443	5	50
Object detector	164	2	5
Total	13,245 (25.55%)	104 (54.16%)	163 (16.77%)

**Table 2:** Comparison of the proposed GMM-based background generator and previous research results.

Target FPGA	Circuit	LUT	Slice	DSP48s
Virtex5	Proposed	729	325	3
	[35]	1066	346	10
	[36]	724	323	3
Virtex6	Proposed	794	352	3
	[36]	788	349	3

**Table 3:** Comparison of the processing speed of our approach with other work.

Image Size	Processing Speed (fps)	
	Fast MOD [23]	Proposed
480 × 704	14.8	27.2
368 × 580	22.7	43.1
340 × 570	24.6	47.5
240 × 320	51.2	119.3

**Table 4:** MOD performance comparison between the proposed moving object detector and other algorithms.

Algorithm	Precision	Recall	F-Measure
Rank-constrained 1 [19]	0.95	0.92	0.9348
Rank-constrained 2 [20]	0.83	0.99	0.9030
Kim et al. [29]	0.98	0.78	0.8686
Proposed	0.98	0.95	0.9648
